# Spatial and temporal behavior of rocky mountain spotted fever in Sinaloa, Mexico: study period 2015-2023

**DOI:** 10.17843/rpmesp.2025.422.14236

**Published:** 2025-06-11

**Authors:** Ámbar Castellanos-Tamayo, Nallely Rivero-Perez, Benjamín Valladares-Carranza, Carla Rosenfel-Miranda, Yesica Morales-Ubaldo, José Esteban Aparicio-Burgos, Carolina G. Sosa-Gutiérrez, Adrian Zaragoza-Bastida

**Affiliations:** 1 Academic Area of Veterinary Medicine and Animal Husbandry, Institute of Agricultural Sciences, Autonomous University of the State of Hidalgo, Hidalgo, Mexico. Universidad Autónoma del Estado de Hidalgo Academic Area of Veterinary Medicine and Animal Husbandry Institute of Agricultural Sciences Autonomous University of the State of Hidalgo Hidalgo Mexico; 2 Faculty of Veterinary Medicine and Animal Husbandry, Autonomous University of the State of Mexico, Toluca, Mexico. Autonomous University of the State of Mexico Faculty of Veterinary Medicine and Animal Husbandry Autonomous University of the State of Mexico Toluca Mexico; 3 Faculty of Veterinary Sciences, Austral University of Chile, Valdivia, Chile. Austral University of Chile Faculty of Veterinary Sciences Austral University of Chile Valdivia Chile; 4 Apan Higher School, Autonomous University of the State of Hidalgo, Hidalgo, Mexico. Autonomous University of the State of Hidalgo Apan Higher School Autonomous University of the State of Hidalgo Hidalgo Mexico

**Keywords:** Rickettsia rickettsii, Epidemiology, Cross-sectional Studies

## Abstract

Rocky Mountain spotted fever (RMSF) is a disease caused by *Rickettsia rickettsii*, whose vector and natural reservoir is the tick. However, dogs play an important role in the transmission of this disease. This study aimed to determine the spatial and temporal behavior of RMSF in the state of Sinaloa, Mexico. Information was obtained from the RMSF cases reported during the period 2015-2023, and the temporal and spatial behavior was determined using an endemic channel and the SCAN statistic. The highest RMSF prevalence rate was determined in Escuinapa with 9.6 cases per 10,000 inhabitants. Regarding temporal behavior, three endemic peaks were detected in March, May, and July, and three RMSF clusters were identified, with the main cluster located in the municipality of Ahome with a relative risk of 4.9.

## INTRODUCTION

Rocky Mountain spotted fever (RMSF) is an infectious disease transmitted by vectors, the etiological agent being *Rickettsia rickettsii*^1^. RMSF is a global public health problem and causes more human deaths than other tick-borne diseases in North America. In 2015, the Mexican Ministry of Health declared an epidemiological emergency for RMSF, which affected approximately 4,000 people ^2^. In Sinaloa, there have been reports of RMSF outbreaks in the municipalities of El Fuerte and Choix, with a higher incidence between March and October and a positive association with housing conditions and dogs infested with ticks ^3^.

Ticks are the main vector. The genera *Rhipicephalus* spp., *Amblyomma* spp., and *Dermacentor* spp. are the most important in Mexico; however, recent epidemics in Arizona (USA) and Sonora (Mexico) have been associated with the brown dog tick (*Rhipicephalus sanguineus*). Dogs shorten the distance between infected ticks and humans, who are accidental hosts ^4^^,^^5^. Dogs infested with *R. sanguineu* have been reported in urban and rural areas, as well as inside homes, especially those in poor and illiterate conditions ^6^.

Globalization and climate change impact the epidemiology of vector-borne diseases, such as RMSF, posing a threat to public health and a challenge for establishing prevention and control strategies ^7^. Based on the above, it is crucial to understand the epidemiological behavior of these diseases, as they represent a public health problem. Therefore, this study aimed to determine the spatial and temporal behavior of RMSF in the state of Sinaloa, Mexico.

**KEY MESSAGES**

Motivation for the study. Vector-borne diseases such as Rocky Mountain spotted fever require constant monitoring of their spatial and temporal distribution patterns due to current climate change and atypical conditions.

Main findings. Rocky Mountain spotted fever occurs in Sinaloa throughout the year, with endemic peaks during March, May, and July. In the municipality of Badiraguato, a relative risk of 6.89 was determined.

Implications for public health. The results of this study show that there are municipalities in the state of Sinaloa with a higher risk of contracting Rocky Mountain spotted fever, so the Sinaloa Ministry of Health could implement prevention and health education programs to reduce the risk in these municipalities.

## THE STUDY

### Study area

The study was located in Sinaloa, northwestern Mexico (22°31′ and 26°56′ N, 105°24′ and 109°27′ W), bordering Sonora, Chihuahua, Durango, Nayarit, and the Gulf of California. The average minimum temperature is 10.5 °C in January and the maximum is 36 °C from May to July. Rainfall occurs in summer, from July to September ^8^.

### Type of study

We conducted a retrospective cross-sectional epidemiological study, considering the variables of time and space.

### Data collection

All positive cases from the databases of the Sinaloa Health Secretariat Surveillance System were included, excluding incomplete data to ensure the validity of the results.

*Cases of* FMMR

Information on cases of RMSF in humans was collected during the period 2015-2023 from the historical records of the Epidemiological Surveillance Department of the Health Services of the Sinaloa Ministry of Health ^9^.

*Population data*

To calculate the prevalence rate and spatial and temporal distribution of RMSF, the population of each municipality in Sinaloa was consulted through the 2020 Population and Housing Census of the National Institute of Statistics and Geography (INEGI) ^10^, using only information from 2020.

*Geographic location data*

For spatial analysis of the data, we used the municipality where the RMSF case was reported as the geographic unit. Georeferencing was obtained from INEGI using the latitude-longitude projection system ^11^.

### Spatial and temporal analysis

The RMSF prevalence rate per 10,000 inhabitants was calculated for each municipality for the period 2015-2023 using the following formula:


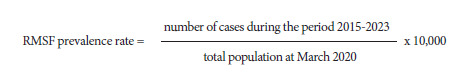


RMSF prevalence rate = number of cases during the period 2015-2023/total population in March 2020 x 10,000

The data obtained from the proportion described above were imported into ArcView GIS 3.2, which already contained information on the municipalities of the state of Sinaloa, to which we assigned a color scheme using a quantile classification method. The number of classes was adjusted according to the data distribution ^12^.

An endemic channel for Sinaloa was created using the quartile method described by Bortman to determine the temporal behavior of the disease ^13^. Using data from positive RMSF cases from 2015 to 2023, we calculated quartiles for each year and plotted graphically in Microsoft Excel®.

In order to determine the spatial distribution of RMSF, we used the SCAN statistic to detect significant clusters. Using information on positive cases, population, and coordinates by municipality in Sinaloa, a retrospective spatial-temporal study was conducted based on the discrete Poisson probability model, assuming that the number of RMSF cases in the geographical area are distributed according to Poisson and under the null hypothesis that the number of expected cases is proportional to the population size ^14^.

The size of the spatial cluster was set at 50% of the population at risk with a minimum of two cases, while the minimum size of the temporal cluster was one year and the maximum size was 50% of the study period. We used 999 Monte Carlo replicates to determine the significance of the clusters, with p-values ≤0.05 indicating a statistically significant grouping ^14^^-^^15^. The graphical representation was made with ArcMap 10.8.

## RESULTS

The RMSF in Sinaloa showed irregular behavior between 2015 and 2023. During this period, 904 cases were reported, with an annual average of 100 cases. The highest number was recorded in 2019, with 230 cases, and the lowest in 2023, with 7 cases. The highest frequency among municipalities was found in Ahome (304 cases), followed by Mazatlán (266 cases) and Culiacán, the state capital (145 cases). The rest of the municipalities reported 60 cases or fewer during the study period.

**Figure 1:**
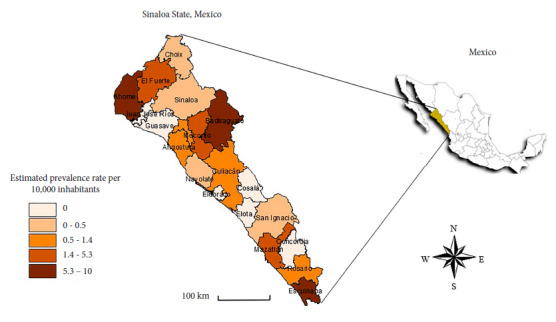
Prevalence rate of Rocky Mountain spotted fever during the period 2015-2023, in the state of Sinaloa, Mexico.

Regarding the prevalence rate of RMSF, Escuinapa was the most affected municipality with 10 cases per 10,000 inhabitants, followed by Badiraguato (7.1), Ahome (6.6), Mazatlán (5.3), and El Fuerte (4). The rest of the municipalities had fewer than 2.2 cases, as can be seen in Figure 1.

**Figure 2:**
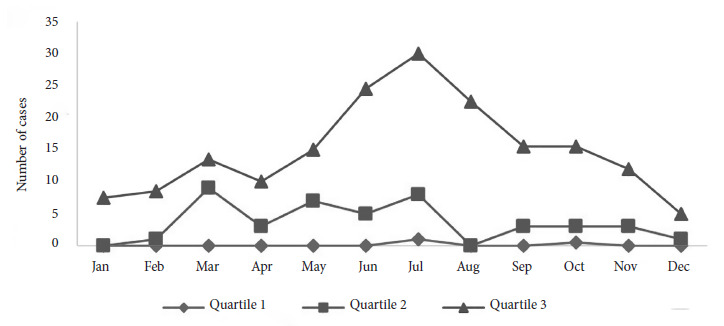
Temporal behavior of Rocky Mountain spotted fever during the period 2015-2023 in the state of Sinaloa, Mexico.

**Figure 3:**
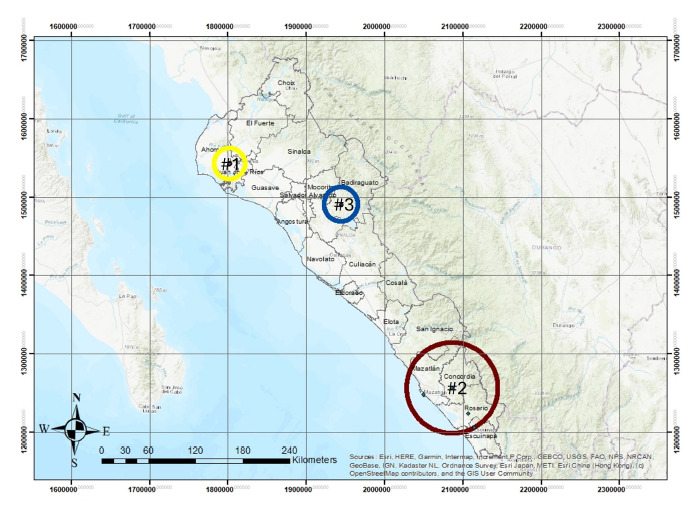
Geographic representation of Rocky Mountain spotted fever clusters during the period 2015-2023, in the state of Sinaloa, Mexico.

We determined, using the endemic index (quartile 2), that RMSF had three endemic peaks in March, May, and July in Sinaloa; however, the disease occurs throughout the year except for January and August, as can be seen in Figure 2. The SCAN spatial statistic identified three clusters in the state of Sinaloa (Figure 3 and Table 1). The first cluster was located in the municipality of Ahome with a logarithmic likelihood ratio (LLR) of 165.5, a relative risk (RR) of 4.91, and 237 observed cases of RMSF compared to 60.97 expected cases.

**Table 1:** Clusters of Rocky Mountain spotted fever during the period 2015-2023, in the state of Sinaloa, Mexico.

Cluster	Location ^a^	Coordinates	Radio (km)	Obs^b^	Exp^c^	RR^d^	LLR^e^	p-value
1	Ahome	25,783 N, 108,994 W	0	237	60.97	4.9	165.5	0.001
2	Concordia, Mazatlán, Rosario y Escuinapa	23,287 N, 106,068 W	58.42	270	84.78	4.1	150.2	0.001
3	Badiraguato	25,362 N, 107,551 W	0	12	1.76	6.9	12.8	0.001

a Municipalities with cases of rickettsiosis, ^b^ number of cases observed, ^c^ number of cases expected, ^d^ relative risk, ^e^ relative probability ratio

The second cluster includes the municipalities of Concordia, Mazatlán, Rosario, and Escuinapa, with an LLR of 150.2 and an RR of 4.12, with 270 cases observed compared to the 84.78 expected cases. Finally, the third cluster was located in the municipality of Badiraguato with an LLR of 12.8 and an RR of 6.89, with 12 cases observed out of the 1.76 expected cases (Figure 3).

## DISCUSSION

RMSF is a significant disease in Mexico, mainly in the north of the country, where fatality rates of up to 40% have been reported ^8^. Children are particularly vulnerable to RMSF due to greater exposure to dogs and ticks through their leisure activities, according to Zazueta et al. ^16^, these activities are a risk factor for RMSF, in addition to environmental and demographic factors.

In Sinaloa, outbreaks of RMSF have been reported in rural areas of El Fuerte and Choix; however, we identified the highest prevalence rate in Escuinapa with 9.6 cases per 10,000 inhabitants, information that does not correspond to the reported RMSF outbreaks ^1^^,^^3^. El Fuerte had a prevalence rate of 3.6 cases per 10,000 inhabitants and Choix 0.3 cases per 10,000 inhabitants, suggesting a change in the endemic behavior of RMSF. Álvarez-Hernández *et al*. ^3^ attributed this to climate change, population displacement, disorderly urban growth, and an increase in pets per household.

RMSF had three endemic peaks in March, May, and July, months with suitable weather conditions. In this regard, Süss *et al*. ^17^^)^ mention that an ambient temperature above 7 °C and relative humidity >85% facilitate the life cycle of ticks. In Sinaloa, RMSF occurred in spring and early summer, consistent with the findings of Chen and Sexton ^18^, who mention that RMSF has a defined seasonal pattern, with epidemic peaks occurring in spring and summer. However, this behavior may vary between geographical regions.

In the United States, the incidence of the disease occurs from April to September, which corresponds to the tick season ^6^, coinciding with the months of May and July reported in this study. In Mexico, in the state of Sonora, increases in RMSF were found from July to November, which do not coincide with those reported by our study, especially considering that the states of Sonora and Sinaloa are neighbors ^19^.

We identified three clusters in our study, suggesting that the location of RMSF clusters may be associated with social or economic factors such as housing hygiene, lack of prophylactic measures such as external deworming of pets, as well as environmental factors that favor the development of the free-living stages of these ticks, thus enabling the tick’s life cycle to be successful ^18^. However, when reviewing the marginalization index, only Badiraguato has a high marginalization index, while the municipalities involved in the clusters have a low or very low marginalization index (Ahome).

Based on the above and assuming the possibility of underreporting of cases in the Sinaloa Epidemiological Surveillance System, it is more likely that spatial clusters are more associated with climatic conditions and arthropod phenology than with sociodemographic factors, a situation similar to that reported by Raghavan *et al*. ^20^^)^ in the United States. There is little information available at this time in the state of Sinaloa to discuss the formation of clusters; however, similar studies conducted in Sonora were unable to determine the formation of clusters ^19^.

The results of this study could be useful for the Sinaloa Ministry of Health to carry out surveillance, prevention, and health education activities in these municipalities, with the aim of breaking the chain of transmission of RMSF. However, future epidemiological studies on RMSF should consider geographic location at the household level, since this study was limited to geographic location at the municipal level. as well as correlating their presence with socioeconomic variables and the use of climate change indices associated with RMSF, since our analyses were limited to a spatiotemporal study without considering the aforementioned variables. Additionally, the possibility of ecological fallacy due to the study design should be considered.

In conclusion, the highest prevalence rate of RMSF was determined in Escuinapa. Regarding temporal behavior, we found three endemic peaks and identified three clusters of RMSF, with the main cluster located in the municipality of Ahome with a moderate risk. However, the secondary clusters located in Badiraguato had a higher risk of developing RMSF according to spatial analysis.
