# Supplementary material for: Spatial and temporal behavior of rocky mountain spotted fever in Sinaloa, Mexico: study period 2015-2023
Source: Rev Peru Med Exp Salud Publica. 2025 Jun 11;42(2):190–5. doi: 10.17843/rpmesp.2025.422.14236 (PMC12377888; doi:10.17843/rpmesp.2025.422.14236)
Supplement: Translation

# Translation: Comportamiento espacial y temporal de la fiebre manchada de las montañas rocosas en Sinaloa, México: periodo de estudio 2015-2023

**Authors:** Ámbar Castellanos-Tamayo, Nallely Rivero-Perez, Benjamín Valladares-Carranza, Carla Rosenfel-Miranda, Yesica Morales-Ubaldo, José Esteban Aparicio-Burgos, Carolina G. Sosa-Gutiérrez, Adrian Zaragoza-Bastida

**Affiliations:** 1 Área Académica de Medicina Veterinaria y Zootecnia, Instituto de Ciencias Agropecuarias, Universidad Autónoma del Estado de Hidalgo, Hidalgo, México.; 2 Facultad de Medicina Veterinaria y Zootecnia, Universidad Autónoma del Estado de México, Toluca, México.; 3 Facultad de Ciencias Veterinarias, Universidad Austral de Chile, Valdivia, Chile.; 4 Escuela Superior de Apan, Universidad Autónoma del Estado de Hidalgo, Hidalgo, México.

**Keywords:** Rickettsia rickettsii, Epidemiología, Estudios Transversales

## Abstract

La fiebre manchada de las Montañas Rocosas (FMMR) es una enfermedad provocada por *Rickettsia rickettsii*, cuyo vector y reservorio natural es la garrapata, sin embargo, los perros son una parte importante en la transmisión de esta enfermedad. El objetivo de la presente investigación fue determinar el comportamiento espacial y temporal de la FMMR en el estado de Sinaloa, México. Se obtuvo información de los casos reportados de FMMR durante el periodo 2015-2023, se determinó el comportamiento temporal y espacial mediante un canal endémico y el estadístico SCAN. La tasa de prevalencia de la FMMR más alta se determinó en Escuinapa con 9,6 casos por cada 10 000 habitantes. Con respecto al comportamiento temporal se observaron tres picos endémicos en los meses de marzo, mayo y julio y se identificaron tres clústeres de FMMR, cuyo clúster principal se ubicó en el municipio de Ahome con un riesgo relativo de 4,9.

## INTRODUCCIÓN

La fiebre manchada de las Montañas Rocosas (FMMR) es una enfermedad infecciosa transmitida por vector, el agente etiológico es *Rickettsia rickettsii*^1^. La FMMR es un problema de salud pública a nivel mundial y causa más muertes humanas que otras enfermedades transmitidas por garrapatas en América del Norte. En el 2015, la Secretaría de Salud de México declaró una emergencia epidemiológica de FMMR que afectó a aproximadamente 4000 personas ^(^^2^. En Sinaloa, existen informes de brotes de FMMR en los municipios de El Fuerte y Choix, observándose una mayor incidencia entre marzo y octubre, y una asociación positiva con condiciones de la vivienda de la población y perros infestados con garrapatas ^3^.

El principal vector de son las garrapatas. Los géneros *Rhipicephalus* spp., *Amblyomma* spp. y *Dermacentor* spp. son los más importantes en México; sin embargo, las epidemias recientes en Arizona (EE. UU.) y Sonora (México) se han asociado con la garrapata del perro café (*Rhipicephalus sanguineus*). Los perros acortan la distancia entre las garrapatas infectadas y los humanos, quienes son hospedadores accidentales ^4^^,^^5^. Se han reportado perros infestados de *R. sanguineu* en zonas urbanas y rurales, así como dentro de las viviendas, sobre todo en aquellas en condiciones de pobreza y analfabetismo ^(^^6^.

La globalización y el cambio climático impactan en la epidemiología de las enfermedades transmitidas por vectores, como la FMMR, lo que representa una amenaza para la salud pública y un desafío para establecer estrategias de prevención y control ^7^. Con base en lo anterior, es crucial entender el comportamiento epidemiológico de estas enfermedades, ya que representan un problema de salud pública. Por ello, el objetivo de la presente investigación fue determinar el comportamiento espacial y temporal de la FMMR en el estado de Sinaloa, México.

**MENSAJE CLAVE**

Motivación para el estudio. Las enfermedades trasmitidas por vector como la fiebre manchada de las Montañas Rocosas requieren de identificar su patrón de distribución espacial y temporal de forma constante por las modificaciones climáticas y atípicas que se presentan en la actualidad.

Hallazgos principales. La fiebre manchada de las Montañas Rocosas se presenta en Sinaloa durante todo el año con picos endémicos durante los meses de marzo, mayo y julio. En el municipio de Badiraguato se determinó un riesgo relativo de 6,89.

Implicaciones para la salud pública. Los resultados de la presente investigación muestran que existen municipios de estado de Sinaloa con mayor riesgo de adquirir la fiebre manchada de las Montañas Rocosas, por lo que la Secretaría de Salud de Sinaloa podría aplicar programas de prevención y educación para la salud para reducir el riesgo en estos municipios.

## EL ESTUDIO

### Área de estudio

El estudio se ubica en Sinaloa, noroeste de México (22°31′ y 26°56′ N, 105°24′ y 109°27′ O), limitando con Sonora, Chihuahua, Durango, Nayarit y el golfo de California. La temperatura promedio mínima en de 10,5 °C en enero y la máxima es de 36 °C de mayo a julio. Las lluvias ocurren en verano, de julio a septiembre ^8^. 

### Tipo de estudio

Se realizó un estudio epidemiológico de tipo transversal retrospectivo, considerando las variables tiempo y espacio.

### Recopilación de datos

Se incluyeron todos los casos positivos registrados en las bases de datos del Sistema de Vigilancia de la Secretaria de Salud de Sinaloa, excluyendo los datos incompletos para asegurar la validez de los resultados.

*Casos de FMMR*

Se recolectó información sobre casos de FMMR en humanos durante el periodo 2015-2023, a partir de los registros históricos del Departamento de Vigilancia Epidemiológica de los Servicios de Salud de la Secretaría de Salud de Sinaloa ^9^.

*Datos de la población*

Para calcular la tasa de prevalencia y la distribución espacial y temporal de la FMMR se consultó la población de cada municipio de Sinaloa a través del Censo de Población y Vivienda 2020 del Instituto Nacional de Estadística y Geografía (INEGI) ^10^, utilizando únicamente la información del año 2020.

*Datos de localización geográfica*

Para el análisis espacial de los datos se utilizó como unidad geográfica el municipio de donde se presentó el caso de FMMR. La georreferencia se obtuvo del INEGI en el sistema de proyección latitud-longitud ^11^.

### Análisis espacial y temporal

Se calculó la tasa de prevalencia de la FMMR por cada 10 000 habitantes durante el periodo 2015-2023 por municipio, la cual se obtuvo mediante la siguiente fórmula:



Los datos obtenidos de la proporción previamente descrita se importaron al programa ArcView GIS 3.2, en el cual ya se había cargado la información de los municipios del estado de Sinaloa, a los que se asignó una coloración graduada mediante un método de clasificación por cuantiles. El número de clases se ajustó de acuerdo con la distribución de los datos ^12^.

Para determinar el comportamiento temporal de la enfermedad, se realizó un canal endémico para Sinaloa siguiendo el método de los cuartiles descrito por Bortman ^13^. Con los datos de los casos positivos de FMMR de 2015-2023, se calcularon los cuartiles para cada año y se representaron gráficamente en el programa Microsoft Excel®..

Para determinar la distribución espacial de la FMMR se utilizó el estadístico SCAN para detectar agrupaciones significativas. Con la información de los casos positivos, población y coordenadas por municipio de Sinaloa, se realizó un estudio retrospectivo espacial-temporal, basado en el modelo discreto de probabilidad de Poisson asumiendo que el número de casos de FMRR en el área geográfica se distribuyen de acuerdo con Poisson y bajo la hipótesis nula de que el número de casos esperados es proporcional al tamaño de la población ^(^^14^.

El tamaño del clúster espacial se estableció en 50% de la población en riesgo con un mínimo de casos de 2, mientras que, el tamaño mínimo del clúster temporal fue de 1 año y el tamaño máximo fue del 50% del periodo de estudio. Se usaron 999 réplicas de Monte Carlo para determinar la significancia de los clústeres, valores de p≤0,05 indicaron un agrupamiento estadísticamente significativo ^14^^-^^15^, la representación gráfica se realizó en el programa ArcMap 10.8.

## RESULTADOS

La FMMR en Sinaloa mostró un comportamiento irregular entre 2015 y 2023. Durante este periodo se reportaron 904 casos, con un promedio anual de 100 casos. El mayor número se registró en 2019, con 230 casos, y el menor en 2023, con 7 casos. En cuanto a la distribución por municipios, la mayor frecuencia se observó en Ahome (304 casos), seguido de Mazatlán (266 casos) y Culiacán, la capital del estado (145 casos). El resto de los municipios presentó 60 casos o menos durante el periodo de estudio.

Con respecto a la tasa de prevalencia de la FMMR, Escuinapa fue el municipio más afectado con 10 casos por 10 000 habitantes, seguido de Badiraguato (7,1), Ahome (6,6), Mazatlán (5,3) y El Fuerte (4). El resto de los municipios presentaron menos de 2,2 casos, como se puede observar en la figura 1.

**Figura 1:** Tasa de prevalencia de la fiebre manchada de las Montañas Rocosas durante el periodo 2015-2023, en el estado de Sinaloa, México.

Con respecto al comportamiento temporal de la FMMR, en Sinaloa, por medio del índice endémico (cuartil 2) se determinó que la FMMR presenta tres picos endémicos en los meses de marzo, mayo y julio; sin embargo, la enfermedad se presenta todo el año con excepción de enero y agosto como se puede observar en la figura 2. El estadístico espacial SCAN, identificó tres clústeres en el estado de Sinaloa (figura 3 y tabla 1). El primer clúster se ubicó en el municipio de Ahome con una relación de probabilidad logarítmica (LLR por sus siglas en inglés), de 165,5 un riesgo relativo (RR) de 4,91 con 237 casos observados de FMMR frente a los 60,97 casos esperados.

**Figura 2:** Comportamiento temporal de la fiebre manchada de las Montañas Rocosas durante el periodo 2015-2023, en el estado de Sinaloa, México.

**Figura 3:** Representación geográfica de los clústeres de la fiebre manchada de las Montañas Rocosas durante el periodo 2015-2023, en el estado de Sinaloa, México.

**Tabla 1:** Clústeres de Fiebre Manchada de las Montañas Rocosas durante el periodo 2015-2023, en el estado de Sinaloa, México.

Clúster	Loc.^a^	Coordenadas	Radio (km)	Obs^b^	Exp^c^	RR^d^	LLR^e^	Valor de p
1	Ahome	25,783 N, 108,994 O	0	237	60,97	4,9	165,5	0,001
2	Concordia, Mazatlán, Rosario y Escuinapa	23,287 N, 106,068 O	58,42	270	84,78	4,1	150,2	0,001
3	Badiraguato	25,362 N, 107,551 O	0	12	1,76	6,9	12,8	0,001

a Municipios con casos de rickettsiosis, ^b^ número de casos observados, ^c^ número de casos esperados, ^d^ riesgo relativo, ^e^ relación de probabilidad logarítmica

El segundo clúster agrupa a los municipios de Concordia, Mazatlán, Rosario y Escuinapa con un LLR de 150,2 y un RR de 4,12 con 270 casos observados respecto a los 84,78 casos esperados. Finalmente, el tercer clúster se ubica en el municipio de Badiraguato con una LLR de 12,8 y un RR de 6,89 con 12 casos observados de los 1,76 casos esperados (figura 3).

## DISCUSIÓN

La FMMR es una enfermedad de importancia en México, principalmente en el norte del país en donde se han reportado tasas de letalidad de hasta el 40% ^8^, los niños son especialmente vulnerables a la FMMR por una mayor exposición a perros y garrapatas por sus actividades de diversión, de acuerdo con Zazueta *et al*. ^16^^)^ estas actividades son un factor de riesgo para que se presente la FMMR, además de los factores ambientales y demográficos.

En Sinaloa, se han reportado brotes de FMMR en zonas rurales de El Fuerte y Choix, sin embargo, en el presente estudio la mayor tasa de prevalencia se identificó en Escuinapa con 9,6 casos por cada 10 000 habitantes, información que no corresponde con los brotes de FMMR reportados ^1^^,^^3^. El Fuerte presentó una tasa de prevalencia de 3,6 casos por cada 10 000 habitantes y Choix 0,3 casos por cada 10 000 habitantes, esto sugiere un cambio en el comportamiento endémico de la FMMR. Al respecto Álvarez-Hernández *et al*. ^3^ atribuyen esto al cambio climático, desplazamiento poblacional, crecimiento urbano desordenado y aumento de mascotas por hogar.

 La FMMR presentó tres picos endémicos en marzo, mayo y julio, meses que presentan las condiciones climatológicas adecuadas, al respecto Süss *et al.*^17^ mencionan que una temperatura ambiental mayor a 7 °C y una humedad relativa >85% facilitan el ciclo de vida de las garrapatas. En Sinaloa, la FMMR se presentó en primavera y en el inicio del verano concordando con lo reportado por Chen y Sexton^18^ quienes mencionan que la FMMR presenta un patrón estacional definido, con picos epidémicos que ocurren en primavera y verano, sin embargo, este comportamiento puede variar entre regiones geográficas.

En Estados Unidos la incidencia de la enfermedad se presenta de abril a septiembre, lo que corresponde con la temporada de garrapatas ^6^, coincidiendo en los meses de mayo y julio reportados en el presente estudio. En México, en el estado de Sonora se observó que los incrementos de FMMR ocurren en los meses de julio a noviembre, resultados que no coinciden con los reportados en el presente estudio, mas aún cuando los estados de Sonora y Sinaloa son vecinos ^19^.

En el presente estudio se identificaron tres clústeres, sugiriendo que la localización de los clústeres de FMMR que pueden estar asociados a factores sociales o económicos tales como la higiene de las viviendas, la falta de medidas profilácticas como la desparasitación externa de las mascotas, así como a factores ambientales que favorecen el desarrollo de los estados de vida libre de estas garrapatas, permitiendo asi el éxito del ciclo de vida de la garrapata ^18^. Sin embargo, al revisar el índice de marginación, solo Badiraguato presenta un índice de marginalidad alto, mientras que los municipios involucrados en los clústeres presentan un índice de marginalidad bajo o muy bajo (Ahome).

De acuerdo con lo descrito en los párrafos anteriores y asumiendo la posibilidad de subnotificación de casos en el Sistema de Vigilancia Epidemiologia de Sinaloa es más probable que las agrupaciones espaciales estén más asociadas a las condiciones climáticas y la fenología de los artrópodos que los factores sociodemográficos, situación similar a la reportada por Raghavan *et al*^20^ en Estados Unidos. Existe poca información hasta el momento en el estado de Sinaloa para discutir la formación de los clústeres, sin embargo, estudios similares realizados en Sonora no lograron determinar la formación de clústeres ^19^.

Los resultados de la presente investigación podrían ser útiles para que la Secretaría de Salud de Sinaloa para que se realicen actividades de vigilancia, prevención y educación para la salud en estos municipios, con el objetivo de romper la cadena de transmisión de la FMMR. Sin embargo, los estudios epidemiológicos futuros sobre la FMMR deberían considerar la localización geográfica a nivel de domicilio, debido a que en la presente investigación se tuvo la limitación en la localización geográfica a nivel de municipio, así como correlacionar la presencia de estos con variables socioeconómicas y el uso de índices de cambio climático asociados a la FMMR, debido a que en los análisis realizados se limitaron a un estudio espacio-temporal sin considerar las variables antes mencionadas. Adicionalmente, se debe considerar la posibilidad de falacia ecológica debido al diseño del estudio.

En conclusión, la tasa de prevalencia de la FMMR más alta se determinó en Escuinapa. Con respecto al comportamiento temporal se observaron tres picos endémicos y se identificaron tres clústeres de FMMR, cuyo clúster principal se ubicó en el municipio de Ahome con un riesgo moderado, sin embargo, los clústeres secundarios ubicados en Badiraguato presentaron más de riesgo de desarrollar FMMR de acuerdo con el análisis espacial.
